# The role of eicosanoids in experimental Lyme arthritis

**DOI:** 10.3389/fcimb.2014.00069

**Published:** 2014-05-28

**Authors:** Carmela L. Pratt, Charles R. Brown

**Affiliations:** Department of Veterinary Pathobiology, University of MissouriColumbia, MO, USA

**Keywords:** *Borrelia burgdorferi*, Lyme arthritis, eicosanoids, resolution of inflammation, inflammation

## Abstract

Experimental Lyme arthritis is an inflammatory arthritis caused by infection of mice with the spirochete, *Borrelia burgdorferi*. It recapitulates many of the disease parameters seen in human patients with Lyme arthritis, and thus serves as a model system for the investigation of disease pathogenesis. While much progress has been made in defining components of the immune response to *Borrelia* infection, an overall understanding of the host response leading to arthritis resistance or susceptibility remains elusive. In this review, we will focus on recent advancements of our understanding of the roles of eicosanoids as inflammatory mediators in the regulation of experimental Lyme arthritis. Eicosanoids, such as PGE_2_ and LTB_4_, are powerful regulators of inflammatory responses and thus may be important mediators of Lyme arthritis.

## Introduction

Lyme disease is a major source of morbidity due to the high incidence of rheumatic, cardiovascular, and neurologic complications that follow infection with the etiologic agent, *Borrelia burgdorferi* (Barbour and Fish, 1993). *B. burgdorferi* is transmitted to the mammalian host through the bite of an infected *Ixodes* tick (Burgdorfer et al., 1982). Acute disease is characterized by a classic enlarging bulls-eye rash called erythema migrans, which resolves on its own if left untreated (Steere et al., 2004). Twenty percent of individuals, despite being infected, will remain disease free following resolution of their skin rash. If not treated with antibiotics at this stage, however, most infected individuals will go on to develop secondary complications including carditis, arthritis, or neurological disease (Steere et al., 2004). Subsets of individuals who receive appropriate antibiotic therapy still develop recurrent episodes of chronic joint inflammation up to years after receiving appropriate treatment (Steere et al., 2004; Iliopoulou and Huber, 2010). The genetic components and/or immune parameters that predispose individuals to develop chronic symptoms associated with Lyme disease or to remain disease free are unclear and the subject of ongoing research (Steere et al., 2004).

Experimental Lyme arthritis is the murine model system of Lyme arthritis and recapitulates many of the disease parameters seen in patients with Lyme arthritis. The murine model is an inflammatory arthritis and requires the presence of live spirochetes within the joint for disease development. Arthritis development, however, is genetically controlled resulting in Lyme arthritis-resistant and -susceptible mouse strains (Barthold et al., 1990). C57BL/6 (B6) mice are the most commonly used Lyme arthritis-resistant strain, while C3H/He (C3H) mice are the most commonly used Lyme arthritis-susceptible strain. Infection of susceptible mouse strains with *B. burgdorferi* results in the development of arthritis which peaks around 3–4 weeks post-infection, and then spontaneously resolves over the next few weeks (Barthold et al., 1996). While live spirochetes are required for disease development, their absolute numbers within the joint do not correlate with arthritis severity. Lyme arthritis-resistant and -susceptible mouse strains can harbor equivalent numbers of spirochetes within their joints, yet maintain their distinct disease phenotypes (Brown and Reiner, 1998; Ma et al., 1998). This defines experimental Lyme arthritis as an immunopathology. Disease development in mice is driven primarily by innate immunity, since arthritis-susceptible mice devoid of T and B cells retain their disease susceptibility (Schaible et al., 1989; Brown and Reiner, 1999). Arthritis resolution, on the other hand, appears to be mediated by the production of anti-*Borrelia* antibodies and spirochete clearance from the joints (Barthold et al., 1996). While infection of mice with *B*. *burgdorferi* is a useful model for studying disease pathogenesis, it is currently unclear if similar disease mechanisms are operational during the immune response to *B. burgdorferi* infection in humans.

## Eicosanoids in Lyme arthritis

**Figure 1:**
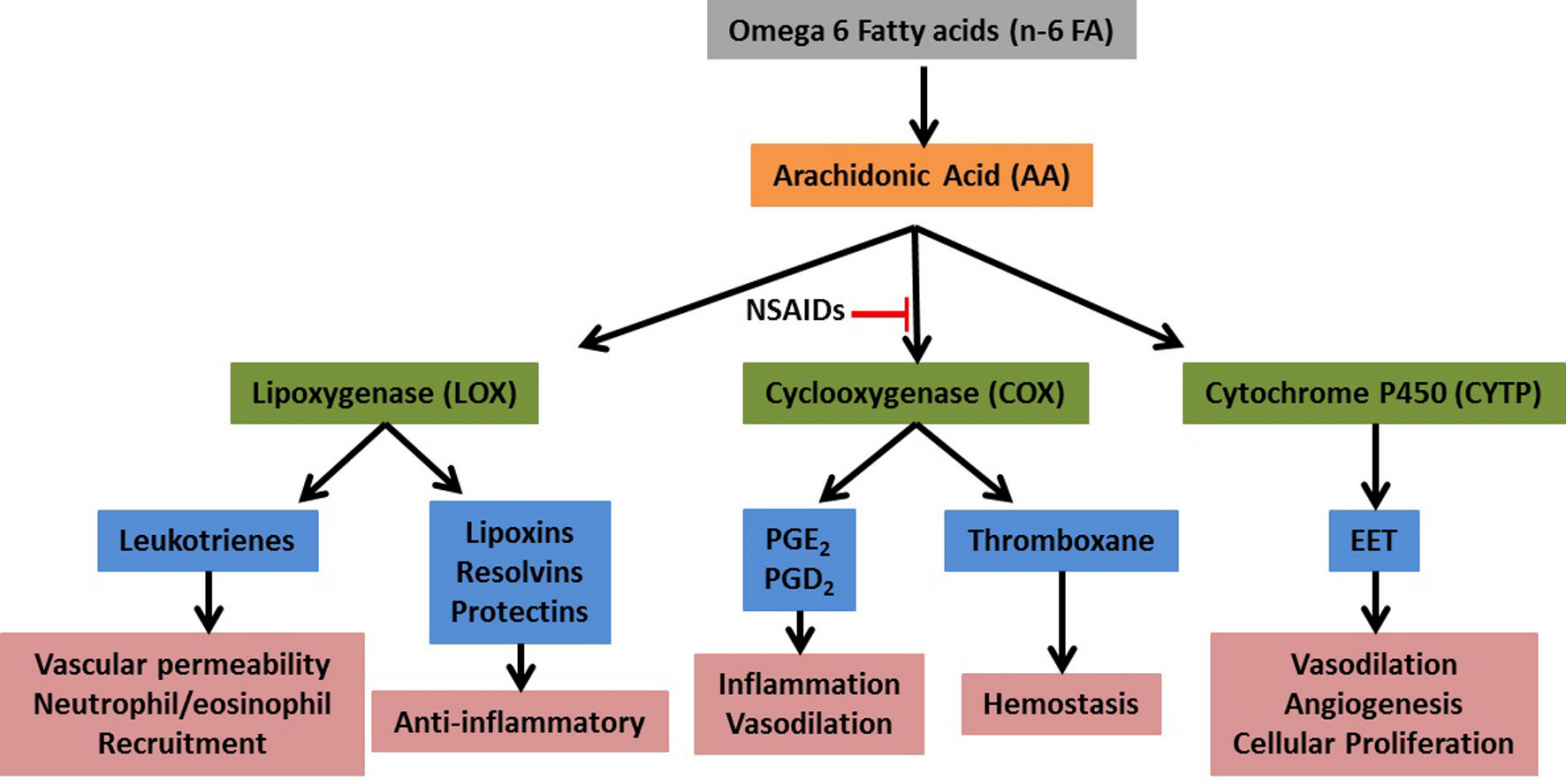
**Simplified eicosanoid metabolic pathway**. Upon tissue damage or infection arachidonic acid (AA) is released from membrane stores by the activity of cytosolic phospholipase 2. The free AA is then acted upon by the primary metabolic enzymes (green) and converted to numerous bioactive compounds (blue).

Eicosanoids are 20-carbon fatty acids derived from the metabolism of arachidonic acid (AA) and are powerful mediators of inflammation (Stables and Gilroy, 2011). Upon activation of immune cells, AA is released from cellular membrane stores primarily via the activity of cytosolic phospholipase A2 (cPLA_2_). The released AA is then metabolized to various biological mediators via three primary enzymatic pathways: cyclooxygenase (COX), lipoxygenase (LOX), and cytochrome P450 (CYTP) (see Figure 1). Each pathway contains additional metabolite-specific enzymatic steps resulting in a wide variety of bioactive compounds (e.g., prostaglandins, leukotrienes, etc). Not all inflammatory cells express all three pathways and there is considerable variation in the production of specific metabolites. In addition, there appears to be a predisposition for certain cells to produce specific metabolites, e.g., macrophages tend to make high levels of prostaglandin (PG)E_2_ while neutrophils tend to produce high levels of leukotriene (LT)B_4_, although they are each capable of making both of these metabolites when stimulated under certain conditions *in vitro* (Kihara et al., 2014). Although eicosanoids are powerful regulators of inflammation, in general their role in mediating an immune response to infection is incompletely understood.

Since experimental Lyme arthritis is an inflammatory arthritis that develops and then spontaneously resolves, it is an ideal model to study how eicosanoids regulate the induction and resolution of an inflammatory response. COX-2 is an inducible gene expressed primarily in immune cells and is responsible for the production of PG during an inflammatory response (Mitchell et al., 1993). Non-steroidal anti-inflammatory drugs (NSAIDs, e.g., aspirin, ibuprofen) are powerful inhibitors of the COX enzymes, with newer drugs (e.g., Celebrex) preferentially inhibiting COX-2. In an initial study of the role of COX-2 in the host immune response to *B. burgdorferi* infection, the expression of COX-2 was found to increase in the joints of infected C3H mice by day 14 post-infection and remain elevated through day 60 (Anguita et al., 2002). Furthermore, treatment of infected mice with a COX-2-specific inhibitor, MF-tricyclic, decreased arthritis severity scores compared to untreated controls at day 14 post-infection without altering T or B cell responses. These results suggested that NSAIDs or other COX-2-specific inhibitors might be effective treatments for Lyme arthritis, possibly without compromising spirochete clearance. We followed up on these results and conducted experiments using two commercially available COX-2-specific inhibitors, rofecoxib (Vioxx) and celecoxib (Celebrex), as well as C3H COX-2 knockout mice (Blaho et al., 2008). In contrast to the previous study, we found no effect of COX-2 deletion or inhibition on the development of experimental Lyme arthritis at a number of time-points throughout the infection. We did, however, find a significant delay or inhibition of arthritis resolution in mice devoid of COX-2 activity. In agreement with the previous study, we found that COX-2 inhibition or deletion had little effect on *Borrelia*-specific antibody production, and clearance of spirochetes from tissues appeared to occur at the same rate in COX-2 deficient and control mice. This demonstrated that arthritis resolution could be uncoupled from spirochete clearance from the joint, and may have important implications for patients with persistent arthritis despite seemingly effective antibiotic therapy. In addition, NSAID therapy to counteract joint pain and swelling during acute Lyme arthritis may be contraindicated and lead to a prolongation of arthritis symptoms. Only a single time point (14 days post-infection) was reported by Anguita et al. which suggested a delay in arthritis development. We did not see a delay in arthritis development in our study, but this may be due to differences in intradermal vs. footpad routes of infection. Further work in this area is required to define the roles of COX-2 metabolites on the development and resolution of experimental Lyme arthritis.

COX-2 is an inducible enzyme that when activated, produces metabolites that are responsible for inciting inflammation and vasodilation (Stables and Gilroy, 2011). The most prominent of these metabolites are PGE_2_ and PGD_2_. Patients diagnosed with arthritis, infectious or immune mediated, are frequently treated with NSAIDs. These drugs are designed to be COX-2 specific to combat the inflammation associated with the activation of the COX pathway. In the experimental Lyme arthritis model, inhibition of COX-2 through gene deletion resulted in milder ankle swelling and arthritis that failed to resolve (Blaho et al., 2008). Histologically, neutrophilic infiltration was still present to the same degree in the COX-2^−/−^ mice as in their wild-type counterparts. PGE_2_ and PGD_2_ levels in the tissue of uninfected and infected COX-2^−/−^ mice were decreased, as expected. Since PGs are responsible for vascular tone, this could explain the attenuation of swelling resulting from their failure to vasodilate to the same degree as wild-type mice. Infected COX-2^−/−^ mice also had unexpected decreased levels of 5-LOX metabolites, potentially contributing to the prolonged inflammatory response seen in these mice (as discussed in the following paragraph).

We have also completed a study of the role of 5-LOX in the development of experimental Lyme arthritis (Blaho et al., 2011). This enzyme is primarily expressed in neutrophils and catalyzes the conversion of AA to LTB_4_ and the cysteinyl leukotrienes (LTC_4_, D_4_, and E_4_). LTB_4_ is a powerful neutrophil chemoattractant and plays an important role in the initiation of arthritis in the K/BxN serum transfer model (Kim et al., 2006), and in collagen-induced arthritis (CIA) (Shao et al., 2006). Both K/BxN serum transfer and CIA arthritis models are experimental autoimmune models of rheumatoid arthritis. Serum from K/BxN mice contain autoantibodies against glucose 6-phosphate isomerase, while the CIA model involves the induction of cross-reactive antibodies to mouse collagen. Both models develop polyarthritis and can be induced in certain mouse strains by the passive transfer of autoimmune serum. Inhibition of 5-LOX activity or inhibition of LTB_4_ signaling through its high-affinity receptor, BLT1, can inhibit the development of arthritis in both of these models. Following infection of C3H mice with *B. burgdorferi*, we found increased expression of mRNA for 5-LOX and its accessory protein, five lipoxygenase activating protein in joint tissue, as well as increased production of LTB_4_ (Blaho et al., 2011). Infection of C3H 5-LOX knockout mice, in contrast to the K/BxN or CIA arthritis models, led to an earlier development of Lyme arthritis. In addition, similar to what was seen in the COX-2 deficient mice, arthritis resolution was delayed or inhibited in the 5-LOX deficient mice as compared to wild type C3H controls. Production of *Borrelia*-specific IgG was decreased in the C3H 5-LOX knockout mice, but spirochete clearance from tissues appeared to be similar to controls, indicating the amount of antibody produced was adequate to mediate spirochete clearance.

**Figure 2:**
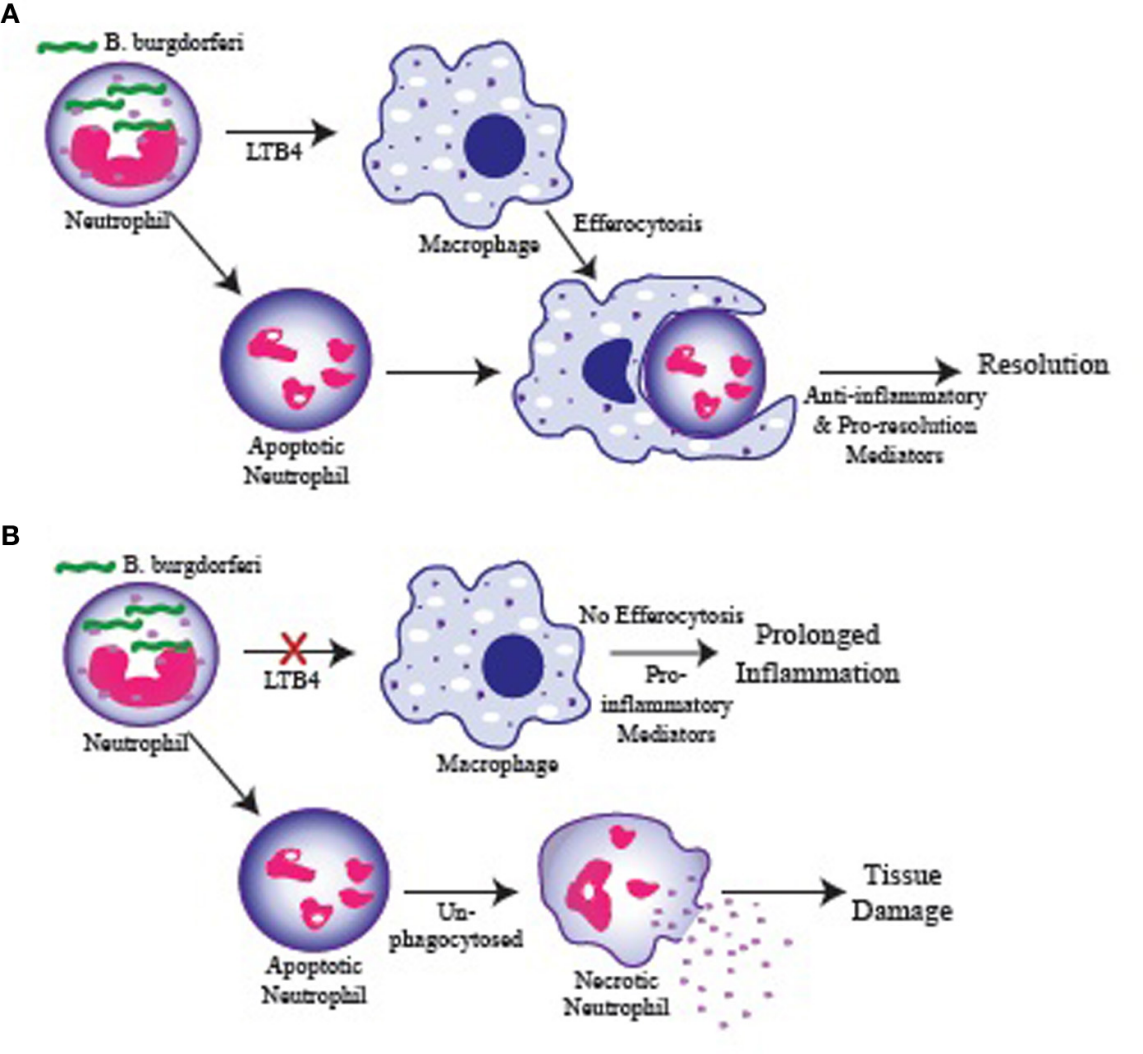
**Possible role for LTB_4_ in regulating resolution of inflammation. (A)** Neutrophils are recruited to the infected joint and engulf *B. burgdorferi*. Neutrophils produce LTB_4_ which stimulate macrophage phagocytosis of apoptotic neutrophils (efferocytosis) and their switch to an anti-inflammatory/pro-resolution phenotype promoting a return to homeostasis. **(B)** Failure of neutrophils to produce LTB_4_ results in inefficient macrophage phagocytosis and failure to clear apoptotic neutrophils in a timely manner. Apoptotic neutrophils in the tissue proceed to necrosis and release their cytoplasmic contents causing tissue damage and promoting continued inflammation. Similarly, macrophages that fail to engulf apoptotic cells remain in a pro-inflammatory state and promote prolonged inflammation.

Products from the 5-LOX metabolic pathway, especially LTB_4_, have been shown to have a significant impact on macrophage phagocytosis and killing of various pathogens (Serezani et al., 2005). Figure 2 depicts how a failure in LTB_4_ production by neutrophils might influence arthritis resolution. *In vitro*, we demonstrated that spirochete uptake and killing by 5-LOX-deficient neutrophils was mostly intact, while 5-LOX-deficient macrophage uptake of *B. burgdorferi* and apoptotic neutrophils was significantly impaired (Blaho et al., 2011). These results suggested that neutrophils may be the primary cells responsible for spirochete clearance, but that macrophage clearance of apoptotic neutrophils may be important for the timely and efficient induction of arthritis resolution and return of the tissue to homeostasis. Further work in this area is required to identify the mechanisms involved, but macrophage uptake of apoptotic cells has been shown to induce their phenotypic change from pro-inflammatory to anti-inflammatory and promote resolution of inflammation (Freire-de-Lima et al., 2006).

## Eicosanoid production during infection

To gain a more complete understanding of the production of eicosanoids in the arthritic joint during *B. burgdorferi* infection, we conducted the first published comprehensive lipidomic study of infected tissue (Blaho et al., 2009a). Additional lipidomic studies using other infectious disease models have since been published, providing further insight into the impact of eicosanoids on immune function and as targets for future therapeutics (Tam, 2013). In our study, joint tissue from arthritis-resistant DBA/2, and -susceptible C3H mice were analyzed for the production of 104 unique lipid species in the eicosanoid metabolome during the development and resolution phases of experimental Lyme arthritis. Several of the eicosanoid pathways between an arthritis-resistant and susceptible strain were different at baseline as well as during *B. burgdorferi* infection, and these differences may drive genetic predispositions to arthritis resistance or susceptibility. Upon infection with *B. burgdorferi*, alterations in the production of metabolites from all three eicosanoid pathways became evident. Specific strain differences noted were elevation in 5,6 epoxyeicosatrienoic acid (EET), and lower PGD_2_ and protectin D1 (PD1) levels in DBA/2 mice as compared to C3H mice. Both strains exhibited elevations in PGE_2_ and a decrease in 11,12 EET. PGE_2_ and PGD_2_ are metabolites derived from the COX pathway and are considered primarily pro-inflammatory. Although PGE_2_ becomes elevated in both strains, PGD_2_ did not significantly increase from basal levels in DBA/2 mice. The lack of elevation of PGD_2_ in arthritis-resistant DBA/2 mice may be one potential factor contributing to the observed strain susceptibility differences.

The CYTP pathway is responsible for producing EETs. The overall function of EETs is vasodilation, angiogenesis, and cellular proliferation (Fang et al., 1996; Pozzi et al., 2005; Blaho et al., 2009a; Stables and Gilroy, 2011). The ratio of 5,6 EET was higher in DBA/2 mice as compared to C3H mice at basal levels and after *B. burgdorferi* infection (Blaho et al., 2009a). 5,6 EET specifically causes vasodilation and is a potent neo-vascular agent (Pozzi et al., 2005). An increase in vessel formation and blood flow at the site of inflammation due to 5,6 EET could improve the clearance of *Borrelia* bacteria in DBA/2 mice as compared to C3H mice, although no differences in bacterial tissue loads were detected. The most significant change within the CYTP pathway in both strains was a decrease in 11,12 EET as compared to other EETs and dihydroxyeicosatrienoic acid (DHET) molecules (Blaho et al., 2009a). Vascular cell adhesion molecule expression is down-regulated in the presence of 11,12 EET in experimentally inflammed murine carotid arteries, which prevented the recruitment of leukocytes to the site of inflammation (Node et al., 1999). It is hypothesized that 11,12 EET acts as an anti-inflammatory agent in a similar fashion to counter balance the other pro-inflammatory mediators in Lyme arthritis, however, more work is needed in this area.

Protectin D1 (PD1) is a product of the metabolism of the omega-3 fatty acid, docosahexaenoic acid (DHA), via the 12/15-LOX pathway. It is associated with anti-inflammatory and pro-resolution properties, including the inhibition of neutrophil influx (Levy et al., 2007; Stables and Gilroy, 2011). In Lyme arthritis, PD1 was significantly elevated in C3H mice, but not in DBA/2 mice (Blaho et al., 2009a). The stable metabolite of PD1 and resolvins, 17-HDoHE, was elevated earlier in infection (day 14) in C3H mice as compared to DBA/2 mice (day 28). Based on this experimental finding, we hypothesize that C3H mice may have a higher threshold of tolerance for PD1, requiring higher levels of PD1 to produce the same effects as in DBA/2 mice. In addition, the susceptibility of C3H mice to Lyme arthritis could be explained by the altered metabolism of up-stream products supported by the appearance of 17-HDoHE earlier in C3H as compared to DBA/2 mice. Further studies are required to determine the exact role these metabolites play in Lyme arthritis resolution.

## Manipulating the eicosanoid profile

Omega-3 polyunsaturated fatty acids (n-3 PUFAs) have received attention for their potential beneficial effects in a vast number of diseases including dermatitis, rheumatoid arthritis, osteoarthritis, neoplasia, cardiovascular disease, and inflammatory bowel disease (Dumlao et al., 2012; Shek et al., 2012). The prominent feature of n-3 PUFAs in these diseases is their anti-inflammatory effects. A diet rich in n-6 PUFAs (e.g., AA) has been linked to the development and persistence of various diseases (Shek et al., 2012). Dietary n-3 PUFAs can compete with AA for incorporation into cellular membranes and for use as substrates for COX and LOX enzymes (Lands et al., 1990). Use of n-3 PUFAs as substrates results in the generation of (n-3) eicosanoids that are generally less potent than analogous (n-6) eicosanoids (Wada et al., 2007). However, the role n-6 and n-3 PUFAs play in the face of infection and how they alter the production of eicosanoids is unclear. There are conflicting reports regarding the benefits of dietary n-3 PUFAs modulating the immune system to aid in the recovery from bacterial pneumonia, while other reports find a detrimental effect during influenza infection (Shek et al., 2012; Sharma et al., 2013). We investigated the effect of dietary n-3 and n-6 PUFAs in murine Lyme arthritis (Dumlao et al., 2012). Feeding *B. burgdorferi*-infected C3H mice a fish oil diet, rich in n-3 PUFAs, shifted the eicosanoid profile toward an anti-inflammatory one, while a soy oil diet, rich in n-6 PUFAs, shifted it toward a pro-inflammatory profile. Again using a lipidomics approach, we found more fatty acid (FA) metabolites identified in fish oil fed mice as compared to soy oil fed mice, with most metabolites being EPA and DHA derived. Based on previous reports, it would be expected that a diet rich in n-3 PUFAs would produce an anti-inflammatory eicosanoid profile. Despite a shift toward anti-inflammatory eicosanoids in mice fed a n-3 PUFA rich diet in Lyme arthritis, there was no effect on the clinical observations or histological findings between the two different diets (Dumlao et al., 2012). It is likely there are several factors involved in the development of arthritis, in addition to the eicosanoid profile. Also, one caveat to this study was that we did not examine the resolution phase of the disease process and thus may have missed the effects of dietary fish oil on arthritis resolution and return to homeostasis.

## Eicosanoid influence on antibody production

Production of *Borrelia*-specific antibodies and clearance of spirochetes from the inflamed joint is thought to mediate the resolution of Lyme arthritis (Barthold et al., 1996). However, as discussed above, C3H mice deficient in COX-2 or 5-LOX developed Lyme arthritis, but failed to resolve their inflammation despite a seemingly effective antibody response and spirochete clearance. Thus, we were interested in taking a closer look at the role eicosanoids might play in antibody responses. Historically the inhibition of prostaglandin production has been linked to decreased antibody responses (Roper et al., 2002). The roles of COX enzymes in antibody production, however, appear to vary depending on the underlying pathology and mode of stimulation. Deletion of the COX-2 gene in a CIA model resulted in decreased production of anti-collagen antibodies and markedly attenuated arthritis development as compared to wild-type and COX-1 knockout mice (Myers et al., 2000). The effect of COX inhibition on antibody production in an infectious scenario has revealed conflicting results. During vaccinia virus infection, deletion of COX-2 resulted in decreased levels of several subclasses of IgG due to failure of immunoglobulin class-switching (Bernard et al., 2010). Similarly, pathogen-specific antibody production was decreased by COX-2 inhibition during *Mycobacterium bovis*-induced arthritis (Turull and Queralt, 2000). More recently, our lab has investigated the effect of COX-2 and COX-1 gene deletion using *B. burgdorferi.* In this infectious model, COX-2^−/−^ mice produced comparable antibody levels to infected wild-type mice. However, infected COX-1^−/−^ mice revealed an inhibition of immunoglobulin class-switching supported by elevated IgM and decreased total IgG levels (Blaho et al., 2009b). In addition, histology revealed defective germinal center formation in COX-1 deficient mice. The prostaglandin, PGE_2_, has been shown to influence the activation and proliferation of lymphocytes, and assist in B cell antibody class-switching (Ryan et al., 2005). We investigated the effect of eicosanoid production when COX-1 was inhibited or deleted *in vitro* and *in vivo.* Deletion of COX-1 decreased the production of PGE_2_ and PGD_2_, although not significantly so as compared to infected wild-type mice in splenic tissue. However, additional eicosanoids that were significantly decreased were TXB_2_, PGF_1α_, PGF_2α_, and PGJ_2_. Inhibition of COX-1 or FP receptor, the receptor for PGF_2α_, in cultures of *B. burgdorferi* infected splenic B cells caused both IgM and IgG production to become significantly decreased as compared to infected controls. This provided evidence that other prostaglandins in addition to PGE_2_ may also be responsible for assisting in antibody production and class-switching. Experiments evaluating the maturation and function of B cells in COX-1 inhibited or deleted mice are needed to further elucidate the mechanism for the failure of immunoglobulin class-switching identified in *B. burgdorferi*-infected mice.

### Conclusion

Lyme arthritis caused by *B. burgdorferi* remains a significant disease in human medicine. The cause for individual susceptibility to chronic arthritis associated with Lyme disease has yet to be explained. The use of murine models of Lyme arthritis has provided significant insight into the pathogenesis and clues into individual susceptibility to chronic disease. It is clear that the immune response and development of Lyme arthritis is complex and multifaceted. Dysregulation of inflammatory and anti-inflammatory mediators, in addition to genetics, add to the factors that determine the ultimate clinical course of disease. Understanding the interaction of cytokines, chemokines, eicosanoids, and genetic influences during *B. burgdorferi* infection will allow improved treatment for individuals with Lyme arthritis.

### Conflict of interest statement

The authors declare that the research was conducted in the absence of any commercial or financial relationships that could be construed as a potential conflict of interest.
